# Supplementary material for: Are carbon emissions trading and green financial instruments synergistic? -Comprehensive quantitative research based on content analysis
Source: PLoS One. 2024 Mar 7;19(3):e0298601. doi: 10.1371/journal.pone.0298601 (PMC10919601; doi:10.1371/journal.pone.0298601)
Supplement: S1 Data — (ZIP) [file pone.0298601.s001.zip › Data/地级市Z_F工作报告.zip!/║■▒▒/╩«╤▀╩╨/└┤╫╘╣1⁄2╓┌║┼í╛┬φ┐╦╩2╛▌═°í┐_2019.docx]

一、2018年工作回顾

　　过去的一年，是十堰加快转型、奋力攻坚的一年。一年来，我们以习近平新时代中国特色社会主义思想和党的十九大精神为指导，以习近平总书记视察湖北重要讲话精神为动力，坚决贯彻省委省政府和市委的决策部署，坚持稳中求进总基调，坚持新发展理念，持续加快转型升级，全力推动高质量发展，迈出了全面建成小康社会的新步伐。

　　——转型发展质效提升。积极应对外部环境复杂多变、实体经济困难增多、汽车市场整体遇冷挑战，我市经济发展稳中育新，转型升级迎难而进。预计地区生产总值增长7%以上，固定资产投资增长11.6%，社会消费品零售总额增长11.5%，地方一般公共预算收入可比增长10.2%。税收收入占地方一般公共预算收入比重71.4%。高新技术产业占GDP比重14.6%。

　　——脱贫攻坚初战告捷。易地扶贫搬迁10.6万人，易迁总体任务基本完成。实现17.06万人脱贫、134个贫困村出列，丹江口市、张湾区、茅箭区可望实现整体摘帽，脱贫攻坚取得阶段性成果。

　　——生态环境总体优化。城区空气质量优良天数305天；全市地表水总体为“优”，丹江口水库水质稳定在Ⅱ类以上，共向北方调水193.6亿立方米。2019年中国生态文明论坛年会选定我市举办。我市被命名为全国“绿水青山就是金山银山”实践创新基地。

　　一年来，我们主要做了以下工作：

　　（一）聚力补短板强弱项，三大攻坚战强力推进。

　　（二）加快转型升级，高质量发展开局良好。

　　（三）狠抓项目投资，新旧动能加快转换。

　　（四）推进乡村振兴，城乡融合发展。

　　（五）深化改革开放，营商环境不断优化。

　　（六）发展社会事业，人民生活持续改善。

　　（七）提升治理水平，社会大局和谐稳定。

　　同时，民族宗教、妇女儿童、外事侨务、气象、档案、粮食、地震、人防、残联、老龄、统计、社科、地方志等各方面工作都取得了新的成绩。

　　二、2019年工作安排

　　今年政府工作的总体要求是：以习近平新时代中国特色社会主义思想为指导，全面贯彻党的十九大和中央经济工作会议、省委第十一届四次全会暨经济工作会议精神，坚持稳中求进工作总基调，坚持新发展理念，推动高质量发展，落实中央“六稳”部署，推进落实汉江生态经济带发展规划，以供给侧结构性改革为主线，深化市场化改革，扩大高水平开放，围绕全省“一芯两带三区”区域和产业战略布局，加快建设“一主四大四新”现代产业体系，继续打好三大攻坚战，统筹推进稳增长、促改革、调结构、惠民生、防风险、强生态工作，保持经济社会持续健康稳定发展，以优异成绩庆祝建市50周年，为全面建成小康社会、建设社会主义现代化强市打下坚实基础。

　　今年发展的主要预期目标是：地区生产总值增长7.5%至8%；规上工业增加值增长7.5%；固定资产投资增长11%；社会消费品零售总额增长11%；地方一般公共预算收入增长8%；城镇常住居民、农村居民人均可支配收入分别增长8%、9%；居民消费价格指数和节能减排控制在省定目标以内。以上目标是基本工作目标，在实际工作中要取得更好成绩。

　　围绕上述目标任务，重点做好八个方面工作：

　　（一）以脱贫攻坚统领经济社会发展全局，坚决打赢脱贫攻坚战

　　——决战决胜整体脱贫摘帽。把脱贫攻坚摆在一切工作的重中之重，坚决克服侥幸、懈怠、拖延、厌战心理，一鼓作气打好脱贫攻坚战，实现整体脱贫摘帽。坚持目标导向、任务导向、效果导向，重点解决好实现“两不愁三保障”“三率一度”面临的突出问题，认真做好丹江口市、张湾区、茅箭区脱贫摘帽迎接国家验收工作；全力实现郧阳区、郧西县、竹山县、竹溪县和房县脱贫摘帽，确保完成17.46万人脱贫、108个村出列任务。加强易地扶贫搬迁后续工作，确保35万易迁对象搬得出、稳得住、能致富。对已脱贫的地方和群众，坚持脱贫不脱钩、脱贫不脱政策、脱贫不脱帮扶，一手抓脱贫，一手防返贫，巩固提升脱贫成果。研究解决那些收入水平略高于建档立卡贫困户的群体缺乏政策支持等新问题。严格落实“三个新增”政策，加快补齐深贫地区短板。大力开展安全饮水、电网改造、村组公路、移动通信、宽带网络、环境改善“六到农家”工程，提升贫困村基础设施和公共服务水平。

　　——做实做深四项重点工作。推进“户户走到”全覆盖，确保无盲区无死角。市县乡三级领导干部巡回走遍所有乡镇、村、户，确保扶贫政策措施落地见效，提升农村群众满意度。建好“扶贫车间”，增强辐射带动作用。深化“千企帮千村”精准扶贫行动，继续抓好香菇、袜业、服装、电子等扶贫产业，把现有的扶贫产业和扶贫车间做强做大做优。加快发展各类扶贫工厂、扶贫车间、扶贫作坊，力争新建扶贫车间（作坊）1900个以上。畅通农产品销售渠道，推进农商互联，扩大电商、供销、邮政等网点覆盖面。推广股份合作、订单帮扶、生产托管等模式，带动每个贫困村至少发展1个农民专业合作社或引进1家龙头企业，带动贫困户年均收入增长15%以上。健全完善市县乡村四级项目库，推动产业项目到村到户到人。强化“安幼养老”，完善社会服务。全面加强兜底保障，重点抓好健康扶贫、教育扶贫，提高基本服务水平。建设一批老年人照料中心、留守儿童活动中心、健康管理中心等农村公共服务设施。持续“强基固本”，凝聚攻坚合力。加强村委会基层组织建设，大力培育脱贫致富带头人，发挥好“领头雁”作用，积极引导全社会更多力量参与脱贫攻坚。

　　——打好脱贫攻坚作风建设持久战。持续深化脱贫攻坚作风建设年活动。狠抓中央扶贫专项巡视、国家和省考核评估等反馈问题整改，深入推进驻村帮扶、资金管理等问题专项整治，强化问题清单管理，立行立改。长短结合、建章立制，构建务实管用的长效机制。加强审计和执纪问责，严肃查处扶贫领域不正之风和腐败问题。重点抓好扶贫领域形式主义、官僚主义和不严不实、弄虚作假等作风问题整治，坚决防止“虚假脱贫”“数字脱贫”“算账式脱贫”，做到扶贫工作务实、脱贫过程扎实、脱贫结果真实。

　　（二）优先发展农业农村，推动乡村全面振兴

　　——加快发展绿色生态有机特色现代农业。推进适度规模经营，深入实施“61”产业强农计划，新建高标准农田8万亩、农业标准化示范基地10万亩。落实粮食安全行政首长责任制，守住粮食安全底线。推进竹房百公里茶叶长廊和汉江百公里林果长廊建设，持续改良柑橘品种，创建茶叶、中药材等特优区。突破性发展农产品精深加工，加快推进丹江口市、郧阳区、房县3个百亿级农产品加工园区建设，壮大农产品加工龙头企业，确保实现农产品加工产值680亿元以上。加强农业品牌建设，积极争创绿色有机品牌，新增“三品一标”15个以上。积极发展新型经营主体，培育农民专业合作社200家、家庭农场200家、专业大户500家。加强服务体系建设，推进生产标准化、营销品牌化，畅通本地农产品进入电商的各个环节。推进农村一二三产业深度融合，大力发展休闲观光农业、乡村旅游和农村电商等新业态。

　　——深化农业农村改革。持续推进农村土地制度改革，加强农村土地确权成果运用，搭建土地流转服务平台，依法规范流转农村土地，积极发展多种形式的适度规模经营。鼓励城市资本、技术和人才向农村集聚，推动城乡要素双向流动，破解项目、产业、资金等难题。推进农村集体产权制度改革，加快农村产权交易市场建设，盘活闲置集体资产，保障农民财产权益。认真开展第三次国土调查、第五次森林资源普查工作。

　　——大力建设美丽乡村。持续推进“四大补短板”工程，加快农村道路、能源、信息、环保等基础设施建设，完善教育、医疗、文化等公共服务，新改建通村公路1400公里，完成2367公里生命安全防护工程，解决安全饮水20万人，改造农村危房7000户。严防严控非洲猪瘟等疫情。学习借鉴浙江省“千万工程”经验做法，深入开展农村环境综合整治、农业面源污染治理，积极推进“厕所革命”，大力发展沼气、太阳能等清洁能源，建成“生态宜居家园”示范村50个以上，努力为老百姓留住鸟语花香的田园风光。

　　——全面发展农村各项事业。加强乡土人才培养，分级分类开展技能培训，大力培育新型职业农民。深入实施“一村多名大学生计划”，选拔培养300名新型农民大学生。繁荣兴盛乡村文化和乡土文明，加大传统村落、历史遗址、民俗文化和民间手工艺保护利用。建设和谐平安乡村，推进移风易俗，完善村规民约，深入开展“十星级农户”“新乡贤”评选活动。健全自治、法治、德治相结合的乡村治理体系。

　　（三）更大力度抓项目投资，加快培育新动能

　　——科学统筹谋划项目。瞄准国家政策取向、产业导向、资金投向，围绕全省“一芯两带三区”战略布局、汉江生态经济带规划、老工业基地搬迁改造、南水北调对口协作、脱贫攻坚、基础设施、社会民生等重点领域，抢抓“十三五”规划中期调整等机遇，谋划重大项目，力争更多项目挤进国家和省政策的“笼子”、规划的“盘子”和资金的“袋子”。积极争取国家专项债券基金、中央和省预算内投资。发挥政府资金牵引作用，多管齐下拓宽投融资渠道，破解多方资本进入工业技改、农业开发、环境治理等难题，构建政府投资、社会融资、民间投资“三轮驱动”新格局，力争民间投资占固定资产投资比重45%以上。谋划好项目储备库，推动好建设库，充实好达效库，力争储备亿元以上重大项目1000个、开工建设亿元以上重大项目400个以上，投资项目“两库”转化率30%以上。

　　——加强和创新招商引资工作。发挥山水、生态、人文、产业优势，坚持引资、引智并举，提高招商引资工作质量。研究国家产业发展趋势，承接先进地区产业转移，促进全市产业升级。突出“一主四大四新”产业链招商，引进一批行业领军型知名企业。瞄准长三角、珠三角、京津冀等重点区域，开展以商招商、小分队招商，依托鄂粤港经贸洽谈、楚商大会、新能源汽车主题峰会等重大招商活动和十堰经济技术开发区、武当山旅游经济特区等平台，挖掘招商潜力，提升招商质效。压紧压实招商引资责任，完善考核督办机制，提高资金到位率、项目开工率和投产率，确保新引进注册规模项目220个以上。

　　——强化服务推进项目。继续实施首席服务官、项目拉练等措施，落实服务专班责任制，确保完成年度1500亿投资任务。推进一批重大基础设施补短板项目，开工建设西十高铁、竹山通用机场等项目，加快推进十巫高速、十淅高速、京能热电2号机组、夹河关水电站、孤山航电枢纽、滨江新区至武当山一级路等在建项目，确保汉十高铁、十堰高铁北站、武当山西站、丹江口南站等竣工运营。

　　（四）持续推进产业转型升级，夯实高质量发展基础

　　——全力推动制造业高质量发展。找准我市在汉随襄十制造业高质量发展带中的位置，加快调整工业结构，构建主导产业支撑有力、新兴产业协同发力的完整工业体系。实现规上工业总产值2300亿元以上，新增规上企业50家，促进主营业务收入利润率和产能利用率有效提升。突出“商乘并举、油电并重”，做强汽车产业。全力服务东风汽车集团有限公司，深化落实“1+5”战略合作协议，加快推进东风装备减速器项目建设，确保东风商用车总装及车架业务优化项目竣工投产，东风小康迁建改造、易捷特新能源电动车、双星东风轮胎4.0新工厂等项目如期量产。深入实施“整车专用化”战略，加快推进驰田金刚10万辆专用车、帕菲特智能特种作业车等项目建设。做强做大汽车零部件产业，推进大洋电机、锂诺新能源电池、东风力神动力电池等配套项目尽快达产。突出技改扩规，提升传统产业。深入落实“万企万亿技改工程”，改造升级轻工纺织、冶金化工等产业，提升技改投资比重，确保技改投资200亿元以上。突出提质增量，壮大新兴产业。大力发展智能制造、信息技术、生物医药、新能源与新材料等新兴产业，重点支持万润新能源、国瑞智能装备、长江星医药、国瓷电子新材等企业发展。

　　——加快发展现代服务业。推进服务业提速升级行动计划，力争完成服务业增加值800亿元以上。坚持全域规划、全要素整合，推进旅游业由单一的门票经济向全产业链融合升级转变。加快编制全域旅游规划、大武当旅游规划和汉江旅游规划，争创国家全域旅游示范区。舞活武当山龙头，建设新区、改造老区、开发湖区、提升景区，发展民宿1000家。支持县市区整合资源、捆绑创建5A级景区，新增3A级以上景区4家以上。提档升级郧西天河水乡、房县黄酒民俗村、竹溪楠木寨、郧阳青龙山等一批景区。开工建设游客集散中心、欢乐世界等项目。力争接待游客7200万人次，实现旅游收入760亿元以上。协同发展传统商贸和现代物流业，改造升级商贸连锁、仓储式购物、一站式商场等零售业态，大力发展社区商业，打造 “15分钟生活圈”。加快推进华西新城、林安综合物流园、鄂西北集疏运配送中心等项目建设。突破性发展新兴服务业，巩固电商进农村综合示范县全覆盖创建成果，力争电商交易额增长25%以上。培育壮大普惠金融、文化创意、信息服务等新业态。

　　——强化创新驱动。建好建强科技创新平台，积极争创国家级高新区，鼓励各地创建省级高新区；推进国家科技成果转化服务示范基地建设。打造“双创”升级版，加大政策支持力度，创建创业创新联盟，提升孵化机构和众创空间服务能力，推动线上线下结合、产学研用协同、大中小企业融合，促进创新成果转化应用。积极培育引进科技领军人才和团队。力争新认定高新技术企业20家，提升高新产业增加值和研发经费占GDP比重。加强技能人才培养，培育更多“车城工匠”和“行业技术能手”。深入推进质量提升行动，继续争创全国质量强市示范城市、长江质量奖，抓好3个国家级公共服务标准化试点创建，组织评选武当质量奖，培育湖北名牌15个。

　　（五）推动新一轮改革开放，塑造发展新优势

　　——巩固深化供给侧结构性改革。落实 “巩固、增强、提升、畅通”要求，用改革的办法和市场化、法治化手段，巩固提升“三去一降一补”成果。加大多元化住房供应力度，促进房地产市场平稳健康发展。优化金融生态环境，提高直接融资比重，创新小微金融服务产品，积极扩大信贷投放规模。重点防范地方政府债务、企业债务风险，有效降低商业银行不良贷款和地方政府债务。

　　——抓紧抓实关键领域改革。深化 “放管服”改革，全面推进“证照分离”“多规合一”，完善投资项目“三个一”审批服务。加快推进“一张网”和数字政府建设，完善智慧政务服务体系，扩大 “一次办好”覆盖面，实现网上可办率不低于70%。深化财税体制改革，突出预算编制绩效管理，优化支出结构，提高财政资金使用效率。深化税收征管体制改革，提高财政收入质量。推进投融资体制改革，规范政府和社会资本合作。完善地方金融监管体制，做优做强地方法人金融机构。把握时间节点，严格规范操作，全面完成机构改革任务。深化国资国企改革，做好经营类事业单位转企改制工作，统筹推进社会民生领域改革。加快现代化市场体系和信用体系建设。

　　——着力构建全面开放新格局。主动融入“一带一路”，大力发展开放型经济，鼓励支持企业参与国际合作，提高实际利用外资水平。加快推广国际贸易“单一窗口”标准版应用，促进外贸拓市场、增份额，扩大汽车及零部件、茶叶、香菇等出口规模。落实京鄂战略合作协议行动方案。扎实推进援疆工作。

　　——毫不动摇发展民营经济。坚持“两个毫不动摇”“三个没有变”，落实我市六大专项行动实施方案。引导金融机构加大信贷投入，拓宽融资途径，健全担保体系，解决融资难融资贵问题。全面落实减税降费政策，出台优惠政策，降低用能、用地、用工、物流、制度性交易等成本，切实减轻企业负担，让企业轻装前进。深化商事制度改革，降低市场准入门槛，建立协同创新体系，全力营造公平竞争环境。大力开展“千名干部进千企”活动，积极解决企业发展困难和历史遗留问题。尊重企业家价值，弘扬企业家精神，壮大企业家队伍，保障企业合法经营，让民营企业和企业家吃下“定心丸”、安心谋发展。

　　（六）加快城乡融合发展，建设区域增长极

　　——推进落实湖北“一芯两带三区”战略布局和汉江生态经济带规划。积极对接“一芯两带三区”战略，谋划争取一批重大项目。抓紧编制推进落实汉江生态经济带发展规划实施方案和重点领域专项规划，加快发展战略性新兴产业、先进制造业、高效生态农业、文化旅游业等产业。以完善重大基础设施为先导，建设环丹江库区生态通道，加快沿汉江国省干线升级改造，提高国省道二级及以上公路比重。加强重要省际通道建设，促进省际互联互通。推进落实湖北长江经济带绿色发展十大战略性举措，建设美丽、畅通、创新、幸福、开放、活力十堰。

　　——竞相发展县域经济。鼓励各县市区争先进位、比拼赶超，走绿色发展、特色发展、错位发展之路。设立县域产业发展引导基金，扶持县域特色产业做强做大。支持丹江口水资源利用及水制品产业深度开发、郧阳区农产品精深加工、郧西县清洁能源、房县生物医药、竹山县电子信息、竹溪县有机农产品及天然饮品、武当山特区健康养生等产业发展。重点扶持绿松石、农产品深加工等7个省级重点产业集群发展壮大。合理开发利用好绿松石资源，提高品牌知名度，做大做强绿松石产业。力争县域工业增加值增长8%以上。

　　——提升城市功能品质。坚持以人民为中心的城市发展理念，增强城市发展的持续性、宜居度、归属感，让人民群众生活更便利、更舒心。推进城镇、土地、交通、林业等规划“多规合一”，合理划定城市开发边界和“三区四线”。实施中心城区改造提升工程，启动中心城区水资源配置项目，加快建设百二河生态修复工程。拓展城市绿色空间，完善生态景观廊道、绿道系统和公园体系。科学运营地下综合管廊。完善城市路网，启动风神大道至高铁北站道路建设，开工建设希望路、江苏路跨火箭路立交、北京路综合改造二期等工程，继续推进武当路复线、三峡路、航空路、火箭路、林荫大道1号线及3号线、京东路延长线等建设，完成江苏路延长线、浙江路跨火箭路立交、北京路跨上海路和重庆路立交等工程。深化智慧交通建设，实施城市道路“微循环”改造，深入开展“路口革命”，鼓励支持社会资本兴建公共停车场，治理交通拥堵、停车难等“城市病”。加快配套建设一批智能充电桩。持续推进老旧小区综合改造、加装电梯等工作，加强物业服务监管，改善群众居住环境。高品质建设滨江新区，启动汉江四桥、杨溪至安阳一级路、南水北调博物馆等项目建设。

　　——加快推进新型城镇化。持续提升城乡公共服务水平，补齐农村交通、电力、供水、通信等基础设施短板，推动城市优质教育、医疗、文化等资源向农村延伸，促进城乡产业、空间、设施、服务全方位融合发展。新建竹房城乡融合发展示范片区9个。加强传统村落、特色村镇保护，支持竹溪汇湾贡茶小镇、郧西上津古镇、房县野人谷生态休闲小镇、竹山麻家渡绿松石小镇建设。加快推进4个省级三产深度融合试点镇、34个农村产业融合发展试点示范村建设，创建国家农村产业融合发展示范园。启动十堰至襄阳卧龙500千伏输电线路、十房及两郧一级路、省道六均路复线工程、天河口汉江大桥建设，推进十堰垭子至大川一级路、兰滩口大桥等项目建设，完成土武一级路建设。

　　（七）以办好中国生态文明论坛为抓手，全域提升环境质量

　　——持续加强生态保护与修复。加强生态红线管控，推进勘界定标工作。编制国土空间规划，制定丹江口库区资源开发利用正负面清单。健全基本农田管护体系，科学划定永久基本农田储备区，守牢耕地红线。坚决保护好汉江生态环境，打好长江大保护十大标志性战役。实施精准灭荒15万亩，人工造林13万亩，退耕还林2.3万亩，义务植树600万株。做好松材线虫病等有害生物防治工作。科学防治地质灾害，全面修复裸露山体，加大石漠化治理、小流域综合治理、矿山地质环境恢复和裸露山体植被恢复力度，治理水土流失面积285平方公里。

　　——系统推进污染防治。坚决打好碧水保卫战，全面落实“河长制”，深入推进河流“清四乱”行动，推进巩固神定河、泗河重点支沟治理，全面消除劣Ⅴ类水体。建立乡镇污水处理厂运行管理长效机制，确保标准化运行、规范化管理。丹江口库区水质稳定保持Ⅱ类标准，全市地表水达到或好于Ⅲ类水体比例94%以上。坚决打赢蓝天保卫战，强化散煤、扬尘、餐饮油烟、秸秆焚烧等重点领域大气污染物管控，积极应对污染天气，确保全市空气质量优良达标天数比例在86%以上。扎实推进净土保卫战，开展土壤污染状况详查，加强未利用地保护，强化土壤污染管控和修复。全域推进畜禽养殖废弃物资源化利用工作。继续推进“四个三重大生态工程”，坚决按期完成中央、省环保督查交办的环境问题整改任务。

　　——创新生态文明体制机制。大力发展节能环保、清洁能源等产业，积极倡导绿色生活、绿色消费和绿色出行，推进绿色低碳发展。全域开展生态示范创建，全力争创国家生态文明建设示范市，支持丹江口市、竹溪县创建国家生态文明建设示范县，郧阳区、竹山县和郧西县创建省级生态文明建设示范县，创建生态乡镇37个、生态村142个。严格执行环保第三方考核和“一票否决”制。继续做好领导干部自然资源资产离任审计、自然资源资产负债表试点工作。精心筹备办好中国生态文明论坛年会。

　　（八）增进民生福祉，增强人民群众获得感

　　——加强就业和社会保障。实施积极的就业政策，多渠道开发就业岗位，推进创业带动就业，新增城镇就业3.6万人，再就业1.8万人，城镇登记失业率控制在4.5%以内。深入推进社保共享计划，努力实现社会保险法定人员全覆盖。积极落实待遇调整政策，稳步提高养老金水平。全面落实社会救助、社会福利、优抚安置等政策，加强农村“三留守”人员和残疾人关爱服务。推进殡葬制度改革。加快市殡仪服务中心、市老年人养护中心、市公共实训基地、7所农村福利院等建设。

　　——办好公平优质教育。普惠发展学前教育，大力扶持公办幼儿园发展，提高普惠性民办幼儿园覆盖率。均衡发展义务教育，持续优化中小学校布局和优质教育资源配置，推进东风与地方学校融合发展，逐步解决 “择校热”“大班额”等问题。推进高中阶段教育普及攻坚计划，稳妥推进新高考改革。支持职业教育打造特色品牌。支持湖北医药学院加快建设国内一流学科。新建车城西路二小、天津路二小、柯家垭小学，改扩建市人民小学、东风21小学，推进湖北汉江技师学院项目建设。

　　——推进健康十堰建设。认真落实“健康十堰2030”行动方案，提高全市人民健康水平。全面深化公立医院综合改革，县域医共体实现全覆盖。进一步完善分级诊疗、现代医院管理、全民医保、药品供应保障、综合监管等制度，推广家庭医生签约服务。支持中医药事业传承发展。落实国家生育政策，促进人口均衡发展。加快推进市妇幼保健院新院区、市中医医院业务综合楼、市西苑医院传染病应急隔离病区、太极湖医院建设。

　　——繁荣发展文化事业。深度挖掘文化资源，加强汉水文化、道教文化研究，培育宣传文化名家，打磨推广文化精品，做大做强文化产业，持续提升文化及相关产业增加值比重。实施文化惠民工程，完善公共文化服务体系。加强文物保护利用和文化遗产保护传承，抓好武当山古建筑群等历史遗迹维修保护工作，积极创建历史文化名城。推进汽车文化遗产保护利用，留住工业“乡愁”。广泛开展全民健身，举办十堰市第六届运动会和第七届世界军人运动会火炬传递活动。加快推进青少年户外培训基地、广电传媒中心项目建设。

　　——全民参与创建全国文明城。大力推进第六届全国文明城市创建工作，持续推进“十大工程”，加速补齐创建短板，提升创建综合实力。深入开展志愿者行动，打造 “一公里志愿服务圈”。巩固提升创建成果，合力推进“五城联创”。开展第七届道德模范评选表彰、学习宣传活动。坚持培育和践行社会主义核心价值观，弘扬时代新风，提升全社会文明程度。

　　——构建共建共治共享社会治理格局。持续深入开展扫黑除恶专项斗争，严厉打击和惩治各类违法犯罪活动，深挖黑恶势力 “保护伞”，严防黑恶势力侵蚀基层政权。推进 “法治十堰”建设，深入开展 “七五”普法，加强信访法治化建设，着力化解信访积案。深化“平安十堰”建设，持续推进 “长安杯”创建，深入实施 “雪亮工程”，健全立体化信息化社会治安防控体系。压实安全生产责任，强化重点行业领域监管，坚决预防和遏制重特大事故发生。完善应急救援体系，提高突发公共事件防范处置能力。落实 “四个最严”要求，加强食品药品安全监管。深化国防动员和双拥共建，巩固军政军民团结。

　　三、着力建设人民满意政府

　　新时代召唤新作为。政府及其组成部门工作人员务必恪尽职守、敬业奉献，全面提升治理服务能力，当好人民满意的公仆。

　　——忠诚担当、务实干事。坚持以习近平新时代中国特色社会主义思想武装头脑，树牢“四个意识”，增强“四个自信”，践行“两个维护”。自觉坚持和落实党对一切工作的领导，坚决贯彻落实党中央国务院大政方针，当好省委省政府和市委决策部署的忠实执行者。强化政治担当、历史担当、责任担当，把对党忠诚、为党分忧、为民造福融入血液、铸入灵魂。坚持“干”字当头，“实”字托底，敢干事、会干事、干成事，讲实话、出实招、求实效，不畏艰难、不惧险阻，敢于担当、善于担当，努力干出无愧于新时代的新业绩。

　　——勤勉尽责、高效干事。聚焦主业主责，强化政府工作人员职业道德、职业操守，坚持守土有责、守土负责、守土尽责，不搞遥控指挥，不当甩手掌柜，承诺的事项坚决兑现，既定的事情坚决办成。不断提升专业精神、专业能力，弥补知识弱项、能力短板、经验盲区，克服本领恐慌，成为行家里手。践行严细深实工作作风，把工作重心下沉到基层，在一线掌握情况，在一线解决问题，在一线推动工作，以钉钉子精神抓好落实。坚持大胆放权、严格监管与优质服务相统一，加快转变政府职能，提高工作效能，优化营商环境。

　　——依法行政、规范干事。严格遵守宪法法律，把法治理念贯穿政府工作全过程，做到依法用权、依规履责、依章办事。认真落实政府会前学法等制度，完善依法科学民主决策机制，推进规范公正文明执法。严格执行市人大及其常委会的决议、决定，自觉接受人大和政协监督，认真办理人大代表建议和政协提案，广泛听取民主党派、工商联、无党派人士和人民团体意见，主动接受社会和舆论监督。加强诚信政府建设，深入推进政务公开，让权力在阳光下运行。

　　——清正廉洁、干净干事。认真履行政府党组全面从严治党主体责任和“一岗双责”，模范带头执行廉洁自律准则，深入推进政府系统党风廉政建设和反腐败斗争。坚决落实中央八项规定精神和省市实施办法，持续加大力度整肃庸政懒政怠政行为，驰而不息纠四风、转作风、正行风。树立过紧日子思想，严控“三公”经费，压缩一般性支出，把每一分钱都花在刀刃上。扎紧制度笼子，强化审计监督，加强工程建设、土地出让、政府采购、产权交易等重点领域监管。坚决查处违法违纪案件和群众身边的腐败问题，打造干部清正、政府清廉、政治清明的良好生态。
